# Structural Analysis of *Bacillus subtilis* Sigma Factors

**DOI:** 10.3390/microorganisms11041077

**Published:** 2023-04-20

**Authors:** Katherine M. Collins, Nicola J. Evans, James H. Torpey, Jonathon M. Harris, Bethany A. Haynes, Amy H. Camp, Rivka L. Isaacson

**Affiliations:** 1 Department of Chemistry, King’s College London, Britannia House, 7 Trinity Street, London SE1 1DB, UK; 2 Department of Biological Sciences, Mount Holyoke College, 50 College Street, South Hadley, MA 01075, USA

**Keywords:** *Bacillus subtilis*, sigma factor, SigE, X-ray crystallography, structure, Alphafold, sporulation

## Abstract

Bacteria use an array of sigma factors to regulate gene expression during different stages of their life cycles. Full-length, atomic-level structures of sigma factors have been challenging to obtain experimentally as a result of their many regions of intrinsic disorder. AlphaFold has now supplied plausible full-length models for most sigma factors. Here we discuss the current understanding of the structures and functions of sigma factors in the model organism, *Bacillus subtilis*, and present an X-ray crystal structure of a region of *B. subtilis* SigE, a sigma factor that plays a critical role in the developmental process of spore formation.

## 1. Introduction

Sigma (σ) factors are bacterial protein modules that plug into RNA polymerase (RNAP) to recruit the enzyme to specific programs of gene transcription via recognition of promoter DNA and the subsequent initiation of transcription [1,2]. The vast majority of σ factors are members of the σ^70^ protein superfamily, which is subdivided into four classes based upon their extent of conservation and the presence/absence of the conserved σ domains (σ1.1, σ2, σ3, and σ4 connected by flexible loop regions) that mediate interactions with RNAP and/or promoter DNA [3]. All bacteria employ an essential primary σ factor (Class I) that directs transcription of housekeeping genes [4]. Many bacteria also possess alternative σ factors (Classes II, III, and IV) that compete for binding to RNAP and redirect it to transcribe sets of genes required for adaptive responses [5]. Hence, the suite of genes expressed in a bacterial cell can be globally reprogrammed simply by manipulating the levels, activity, or availability of alternative σ factors [1].

The model organism, *Bacillus subtilis*, uses a set of well-characterised sigma factors to orchestrate different phases of its lifecycle [6]. As well as interacting with RNA polymerase, sigma factors can bind to many other proteins, including anti-sigma factors which prevent their binding to RNA polymerase in circumstances when their transcription programmes are not required [7]. There are also proteins that compete with sigma factors for binding to the same site on RNAP in another regulatory mechanism [8]. In isolation, sigma factors include several intrinsically disordered regions which allow the domains to wrap around protein partners including RNAP and anti-sigma factors [9]. This flexibility has precluded finding an experimental structure solution for most sigma factors; up until 2002, the only available *Bacillus* sigma factor structure was a stretch of fifty amino acids from SigF, derived from the extremophile *Bacillus stearothermophilus*. This was solved in complex with the anti-sigma factor SpoIIAB (PDB: 1L0O [10]) by X-ray crystallography to 2.9 Å resolution [10]. The first solved fragments of *B. subtilis* sigma factors only emerged in 2017 with domains from SigW (PDB: 5WUQ [11]) and SigA (PDB: 5MWW [12]), as outlined in Table 1. With the refinements supplied by AlphaFold2 [13], the predicted structures of all *B. subtilis* sigma factors are now publicly available in the AlphaFold Protein Structure Database [14].

All but one of the *B. subtilis* sigma factors belong to the σ^70^ factor family, with the only outlier, SigL, being a member of the σ^54^ factor family (see Table 1). In *B. subtilis* there are four sigma factors known to control sporulation—the process in which the bacteria become long-lived dormant spores to survive stress conditions (reviewed in [15,16]). This happens through a genetically choreographed sequence of events in which a cell divides asymmetrically and the smaller cell (forespore) is engulfed by the larger (mother cell), which ultimately lyses after supporting the spore through its metabolic shutdown and building it a sturdy outer shell. The sigma factor “puppeteers” involved in this process are SigF in the forespore and SigE in the mother cell at the early phases; then, these are replaced by SigG and SigK, respectively, as sporulation progresses [17]. Many of the remaining sigma factors are involved in the response to external and environmental conditions (e.g., acid stress), forming the group of extracytoplasmic function (ECF) sigma factors [18].

In earlier work we solved the structure of CsfB/Gin, an anti-sigma factor that acts on both SigG and SigE during sporulation [19,20]. Here we present an experimentally solved X-ray crystal structure of SigE residues 17–133 and review all of the available experimentally-solved and AlphaFold-predicted *B. subtilis* sigma factor structures.

**Table 1:** Sigma factor family members in *B. subtilis* (Data compiled from SubtiWiki [21], PDBe [22], AlphaFold Database [14] and other sources as indicated. For AlphaFold structures σ1 (turquoise), σ2 (slate blue), σ3 (olive), σ4 (raspberry)). Note the unlikely helix prediction for SigI (black).

σ Factor	Molecular Weight (kDa)	Domains & Group	Experimentally Solved *B. subtilis* Structures	Function Summary	AlphaFold Structure Prediction
SigA	42.80	σ1, σ2, σ3, σ4 Group I	σ1.1 NMR 5mww [12] Cryo-EM BmrR transcription activation complex 7ckq [23]	Housekeeping	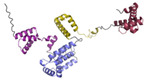
SigB	29.99	σ2, σ3, σ4 Group III	N/A	Stress response [24,25]	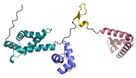
SigD	29.32	σ2, σ3, σ4 Group III	N/A	Chemotaxis & flagellar gene expression [26]. Expression of autolysin [27]	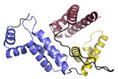
SigE	27.55	σ2, σ3, σ4 Group III	NMR σ2 chimera with GTAAAA 5or5	Early stages of sporulation (Mother cell only) [28]	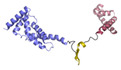
SigF	29.22	σ2, σ3, σ4 Group III	N/A	Early stages of sporulation (Forespore only) [29]	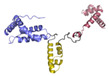
SigG	29.92	σ2, σ3, σ4 Group III	N/A	Late stages of sporulation (Forespore only) [30]	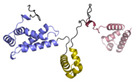
SigH	25.30	σ2, σ3, σ4 Group III	N/A	Expression of genes associated with transition from growth phase to stationary phase. Initiation of sporulation [31]	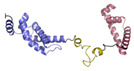
SigI	29.04	σ2, σ3, σ4 Group III	N/A	Heat shock response [32]	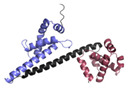
SigK	33.00	σ2, σ3, σ4 Group III	N/A	Late stages of sporulation (Mother cell only) [33]	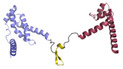
SigL	49.54	σ54 family	N/A	Cold shock response [34]. Assimilation of nitrogen sources/amino acid catabolism [35]	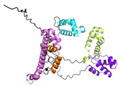
SigM	19.26	σ2, σ4 Group IV	N/A	Extracytoplasmic function (ECF) [36]. Halophilic gene expression [37]	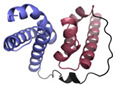
SigV	19.57	σ2, σ4 Group IV	N/A	ECF. Present during outgrowth from endospore, but knockout does not inhibit outgrowth [38]	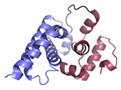
SigW	21.57	σ2, σ4 Group IV	Full length crystal structure with anti-sigma factor RsiW 5wur [11], 5wuq [11]; crystal structure of σ4 bound to DNA 6jhe [39]	ECF. Alkaline shock response [40]	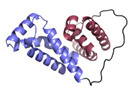
SigX	23.03	σ2, σ4 Group IV	N/A	ECF. Regulation of peptidoglycan synthesis [41]? Modification of cell envelope and resistance to antimicrobial peptides [42].	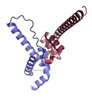
SigY/yxlB	21.21	σ2, σ4 Group IV	N/A	ECF. Production of and resistance to antibiotics (sublancin) through maintenance of Spβ prophage [43].	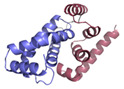
SigZ	20.57	σ2, σ4 Group IV	N/A	ECF	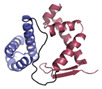
Xpf	19.95	σ2, σ4 Group IV	N/A	Positive control factor (PCF). Linked to PBSX prophage—induces bacterial death in response to DNA damage: “Bacterial suicide” [44]	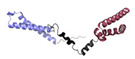
YlaC	20.78	σ2, σ4 Group IV	N/A	ECF. Resistance to oxidative stress [45]	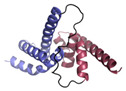
SigO-RsoA	22.54; 9.00	σ2, σ3; σ4 Group III	N/A	Response to acid stress [46]. Expression also induced by antibiotics that target the cell wall	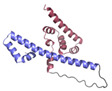

## 2. Materials and Methods

### 2.1. Plasmids and Cloning

The gene for CsfB^A48E^, cloned into bacterial expression plasmid pNIC28 (which adds TEV-cleavable N-terminal His tag), and the SigE^17–239^ in bacterial expression plasmid pET28-TxrA (including His tag, thioredoxin fusion protein and TEV cleavage site) were used as described in [19]. For SigE^17–239^ designed for this study, a BamHI/XhoI-digested PCR fragment covering SigE codons 17–133 was ligated into BamHI/XhoI-digested pET28-TxrA plasmid (described above).

### 2.2. Protein Sequences

SigE^17–239^

MKLGLKSDEVYYIGGSEALPPPLSKDEEQVLLMKLPNGDQAARAILIERNLRLVVYIARKFENTGINIEDLISIGTIGLIKAVNTFNPEKKIKLATYASRCIENEILMYLRRNNKIRSEVSFDEPLNIDWDGNELLLSDVLGTDDDIITKDIEANVDKKLLKKALEQLNEREKQIMELRFGLVGEEEKTQKDVADMMGISQSYISRLEKRIIKRLRKEFNKMV

SigE^17–133^

MKLGLKSDEVYYIGGSEALPPPLSKDEEQVLLMKLPNGDQAARAILIERNLRLVVYIARKFENTGINIEDLISIGTIGLIKAVNTFNPEKKIKLATYASRCIENEILMYLRRNNKIR

CsfB^A48E^

MDETVKLNHTCVICDQEKNRGIHLYTKFICLDCERKVISTSTSDPDYEFYVKKLKSIHTPPLYS

### 2.3. Purification Buffers

Cell lysis buffer: 50 mM HEPES pH 7.5, 300 mM NaCl, 0.5 mM TCEP, 5% glycerol, 5 mM imidazole, 1 mg/mL lysozyme, 10 µg/mL DNaseI, 10 mM MgCl_2_, 2 cOmplete EDTA-free protease inhibitor cocktail tablets, and 2 mM PMSF.

HisTrap Buffer A: 50 mM HEPES pH 7.5, 300 mM NaCl, 0.5 mM TCEP, 5% glycerol, and 10 mM imidazole.

HisTrap Buffer B: 50 mM HEPES pH 7.5, 300 mM NaCl, 0.5 mM TCEP, 5% glycerol, and 250 mM imidazole.

SP Sepharose Buffer A: 50 mM Tris-HCl pH 8.0, 10 mM NaCl, and 0.5 mM TCEP.

SP Sepharose Buffer B: 50 mM Tris-HCl pH 8.0, 1 M NaCl, and 0.5 mM TCEP.

### 2.4. Protein Expression & Purification

All SigE constructs were expressed in the T7 Express *lysY/I^q^ E.coli* strain from New England Biolabs (NEB C3013I). Cells were cultured at 37 °C 220 rpm in an LB growth medium until they reached an OD_600_ of 0.6. Alternatively, for downstream NMR studies SigE was expressed in an M9 Minimal medium supplemented with 0.7 g/L ^15^N-NH_4_Cl, and for carbon experiments also 2 g/L ^13^C-glucose. At OD_600_ 0.6, the cells were induced via the addition of isopropyl β-d-1-thiogalactopyranoside (IPTG) to a final concentration of 0.5 mM. Following induction, the cells were incubated at 22 °C 220 rpm overnight to achieve the expression of SigE. Cells were harvested via centrifugation at 4000× *g* for 30 min and the pellets were snap frozen in liquid nitrogen prior to storage at −80 °C.

All SigE constructs were purified according to the following procedure. Cell pellets derived from 2 L of culture were resuspended in 30 mL lysis buffer. The pellets were thoroughly resuspended and then homogenized via ultrasonication on ice using an 80% amplitude and twelve cycles of 5 s “on” and 25 s “off”. Debris was removed from the lysate by ultracentrifugation at 105,000× *g* for 30 min and passed through a 0.2 µm filter. SigE constructs were purified via immobilized metal affinity chromatography (IMAC). The protein was applied to a HisTrap 5 mL FF column (Cytiva) that had been pre-washed and equilibrated with HisTrap Buffer A. HisTrap Buffer A was passed through the column until the 280 nm trace returned to baseline. At this point the protein was isolated via isocratic elution using the following steps: 4 column volumes (CV) 5% HisTrap Buffer B, 4 CV 10% HisTrap Buffer B, and 4 CV 100% HisTrap Buffer B. Fractions were analysed by SDS-PAGE; those containing SigE as identified through Coomassie staining were dialysed against HisTrap buffer A (containing no imidazole) in the presence of TEV protease overnight at 4 °C. The cleaved SigE was further purified via a reverse Ni-NTA step in which the material was applied to a pre-equilibrated HisTrap 5 mL FF column, although the flow-through was collected. The flow-through was concentrated using a VivaSpin centrifugal concentrator device to <5 mL and applied to a 120 mL Superdex 75 column that had been pre-equilibrated with SP Sepharose Buffer A. Fractions were analysed by SDS-PAGE and then those containing SigE were pooled. Due to the protein clinging to the Vivaspin concentrators at high concentrations, the final concentration step was performed using cation exchange chromatography (SP Sepharose). The protein was applied to a 1 mL HiTrap SP column pre-equilibrated with SP Sepharose Buffer A. The protein was then eluted with 100% SP Sepharose Buffer B into 1 mL fractions. The highest concentration fractions were dialysed against 1 L of the relevant buffer according to downstream usage.

CsfB^A48E^ was produced as described [19]. In short, the protein was expressed in BL21(DE3)pLysS cells using an LB growth medium. Induction was accomplished by adding IPTG to 0.5 mM and incubating at either 37 °C for 4 h or 18 °C overnight. Protein purification was accomplished using IMAC and subsequent SEC.

### 2.5. X-ray Crystallography

All protein preparations were dialysed into 50 mM HEPES pH 7.5, 150 mM NaCl, and 0.5 mM TCEP prior to setting up crystallisation trials.

SigE^17–133^ formed large cuboid crystals in coarse screen condition SaltRx well H7 (0.5 M potassium thiocyanate, 0.1 M Tris pH 8.5) with a protein:liquor ratio of 1:1. These crystals were grown at 7 mg/mL at 16 °C and were discovered after 4 months. The crystals were cryoprotected using 5% glycerol in 3.33 M AmSO_4_. Data were collected at Diamond Light Source beamline I03 at a wavelength of 0.9795 Å with diffraction extending to 2.02 Å. Data were processed in space group C 2 2 21 with the unit cell dimensions: a = 8187, b = 164.94, c = 98.89, α = 90.00, β = 90.00, γ = 90.00. Indexing and integration were carried out using xia2 with DIALS [47], and POINTLESS and AIMLESS were used for the merging and scaling of the data [48]; all of this was conducted on ISpyB [49]. Data were cut to 2.38 Å based upon the CC_1/2_ [50]. The SIMBAD automated pipeline was used to ensure the data did not represent a crystal contaminant [51]. The MrBUMP [52] automated pipeline was used to solve the structure via molecular replacement using PDB entry: 3UGO [53] as a model. Refinement was carried out using Refmac5 [54] with non-crystallographic symmetry (NCS) applied and some automated model building was carried out in Coot [55]. Model building was also aided by PDBredo [56]. Final refinements were carried out in Phenix [57]. The final R_work_ was 0.20 and the final R_free_ was 0.25.

### 2.6. NMR

All protein was dialysed into 50 mM HEPES pH 7.5, 150 mM KCl, and 0.5 mM TCEP prior to NMR data collection.

For chemical shift perturbation studies, ^1^H-^15^N HSQC spectra were collected for 100 μM ^15^N-labelled SigE^17–133^ alone and in the presence of a 2-fold excess CsfB^A48E^. Spectra were collected at 298 K on a 700 MHz Bruker AVANCE NMR spectrometer equipped with a TXI cryoprobe. Incomplete triple resonance datasets were obtained for both 500 μM SigE^17–133^ alone and in complex with CsfB^A48E^ using a 950 MHz spectrometer. All spectrometers were controlled using TopSpin 3. Data processing was performed using NMRPipe [58]/NMRDraw and analyzed using CcpNMR Analysis [59] v2.2.

## 3. Results

### 3.1. Construct Design

**Figure 1:**
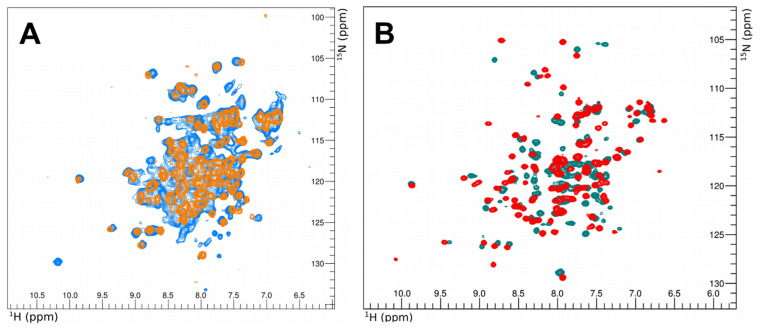
NMR spectra of *B. subtilis* SigE. (**A**) Overlaid ^1^H-^15^N HSQC spectra of ^15^N-labelled SigE^17–239^ (blue) and SigE^17–133^ (orange) constructs. The broad peaks indicate that the former had likely aggregated and/or degraded. The substantial overlap with the peaks of the latter construct, and the lack of many additional peaks, imply that the SigE^17–239^ sample now resembles SigE^17–133^. (**B**) ^1^H-^15^N HSQC spectra of ^15^N-labelled SigE^17-133^ alone (turquoise) and in the presence of a two-fold excess CsfB^A48E^ (red). Chemical shift perturbation clearly indicates interaction between the two proteins.

Initially we produced almost full-length *B. subtilis* SigE (residues 17–239, only missing the initial prosequence that maintains SigE in an inactive state before processing [60]), but we found that it degraded to a smaller domain that remained stable over time, as observed by SDS-PAGE and 2D NMR (Figure 1A). Smaller constructs were designed based on predicted domain boundaries, and SigE^17–133^ was the variant that successfully yielded diffracting crystals.

The first 27 residues of SigE are a pro-sequence that keeps the protein in the inactive state and ensures its localization to the mother cell [61]. These residues get cleaved during sporulation by SpoIIGA to activate the protein. The constructs used in this study lack the first 17 residues as this maintains activity in vivo without requiring processing by SpoIIGA [60].

The A48E mutation of CsfB was utilised as this variant is protected from proteolytic degradation while retaining anti-sigma factor function [19].

#### 3.1.1. SigE^17–133^ NMR

The SigE^17–133^ construct displayed a relatively well-dispersed ^1^H-^15^N HSQC spectrum (Figure 1). Upon titration with unlabelled CsfB^A48E^ (a previously published stability mutant [19]), many chemical shift perturbations were evident, confirming the interaction. Surprisingly, when bound to CsfB, SigE gave rise to better quality spectra despite the increased size of the complex from 13.3 kDa (SigE alone) to 20.8 kDa (13.3 kDa + 7.5 kDa, SigE plus CsfB) (Figure 1B). This is likely in part a consequence of SigE becoming increasingly ordered, leading to greater spectral distribution and reduced peak overlap, as well as possibly improved exchange characteristics. Unfortunately, triple resonance datasets were consistently of poor quality with many peaks missing, so it was not possible to obtain a backbone assignment for SigE^17–133^. Since the complex comprising ^15^N-labelled SigE^17–133^ and CsfB^A48E^ displays sharper peaks than in the HSQC spectrum of isolated SigE^17–133^, we also collected a suite of triple resonance data for the complex on a 950 MHz spectrometer. However, this also proved inadequate for straightforward assignment.

#### 3.1.2. SigE^17–133^ Structure Solution

**Figure 2:**
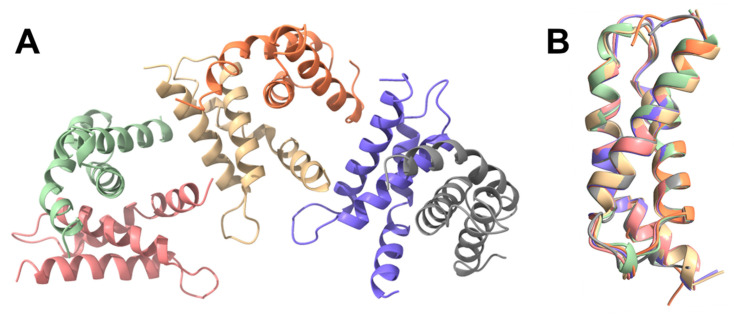
Crystal structure of SigE. (**A**) View of the crystallographic asymmetric unit showing six copies of SigE residues 52–133, each representing a classic helix-turn-helix domain. (**B**) Alignment of the six units of SigE 52–133 from the asymmetric unit. This overlay shows some minor differences between the different biological units (in the same colours as shown in (**A**), with slight structural deviations in the flexible loop regions).

**Figure 3:**
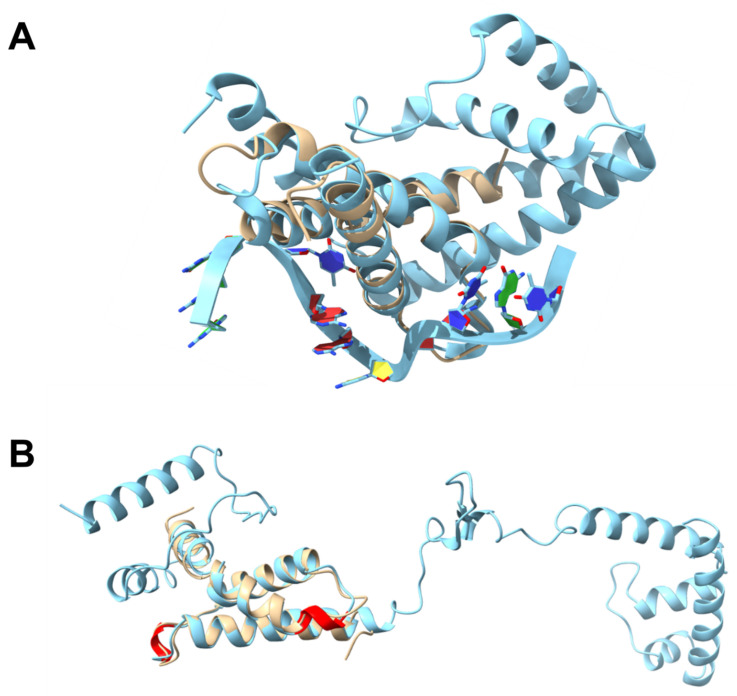
Structural alignments of SigE. (**A**) Structural overlay of SigE^52–133^ crystal structure (cream) with *Thermus aquaticus* RNAP sigma factor A (light blue; PDB: 3UGO [53]) bound to a −10 promoter element ssDNA oligo (TACAAT). The structures align with RMSD: 0.99 over 72 residues, indicating how SigE^52–133^ likely interacts with the −10 promoter in *B. subtilis*. (**B**) SigE^52–133^ crystal structure (cream) overlaid with the AlphaFold model of full-length SigE (light blue) from *B. subtilis* (UniProt ID: P06222). Regions of helix overprediction (residues 77–80, 104–106) by AlphaFold are indicated in red.

Although the SigE^17–133^ construct was used for crystallisation, the structure we obtained (Figure 2A) was an ensemble of six almost identical (overlaying with RMSD from 0.164–0.310 Å over 52–67 atoms; Figure 2B) monomers each comprising residues 52–133, present in the asymmetric unit (deposited with PDB ID: 8B3Z). Crystallographic parameters are shown in Table 2. Crystals grew over a period of 4–6 months and the protein likely lost some N-terminal amino acids during this process. It is also possible that these residues were too flexible to give rise to discernible electron density. The structure is a classic four helix-turn-helix core found in all sigma factor σ2 domains and covers regions σ_2_._1_ (55–78), σ_2_._2_ (79–97), and σ_2_._3_ (98–117) of SigE, which includes the binding sites for both CsfB and the −10 promoter DNA sequence for transcriptional activation (Figure 3A).

Dali searches identified the closest structural homology for *B. subtilis* SigE^52–133^ with *E. coli* RpoS (PDB: 5H6X [62], chain A, RMSD of 0.91 Å) also solved as an isolated domain, and *E. coli* RpoD (PDB: 4ZH3 [63], chain F, RMSD: 0.99 Å) and *M. tuberculosis* SigA (PDB: 6OY5 [64], chain F, RMSD: 1.16 Å) both solved as part of larger holoenzyme complexes. The solved structure aligns very well (RMSD: 0.51 Å) with the AlphaFold prediction for SigE (Figure 3B), with Alphafold providing a slight helix overprediction in the loop between two helices [65].

### 3.2. AlphaFold Prediction of B. Subtilis Sigma Factor Structures

All of the current AlphaFold-predicted structures for isolated *B. subtilis* sigma factors are shown in Table 1. AlphaFold predicts the core structured regions of the sigma factors with high levels of confidence. However, significant regions of the sigma factors are highly flexible in order to accommodate binding to partner proteins. Unsurprisingly, these regions are associated with less confident model building by AlphaFold and often feature unlikely helices [65] (see in particular the prediction for SigI), which is likely a feature of the artificial intelligence being mostly trained on crystal structures.

## 4. Discussion

*B. subtilis* is the best studied Gram positive bacterium and is widely used as a model organism to investigate bacterial cell and developmental biology [16]. Gaining a greater understanding of the processes that underpin genetic regulation in this model system has broader ramifications for antibiotic development and understanding hospital superbugs. In order to do this, however, we require biophysical and structural insight into the behaviour of the multitude of different sigma factors that modulate gene expression [1].

With the advent of AlphaFold, we are now able to access reliable models for the individual domains of most *B. subtilis* sigma factors [14]. The positioning of the connecting loops, especially when wrapped around binding partners in large assemblies, is the next structural frontier, and is well on the way to being cracked both experimentally through large high resolution cryo-EM structures [66] and computationally with AlphaFold multimer [67], which is becoming more and more sophisticated at a rate of knots. These developments are unprecedented given the high flexibility of sigma factors and the difficulty associated with the expression and purification of many of them.

Here, we have presented a crystal structure of the SigE sigma factor from *B. subtilis* and have compared its structure to those others solved experimentally or predicted by AlphaFold. It shares a similar structure with the other members of the σ^70^ family in *B. subtilis*. Whilst there are few experimentally solved structures of the sigma factors and their domains in *B. subtilis*, there is wider coverage of the various domains from sigma factors across bacterial species. These structures, combined with the models from AlphaFold, provide a good overall picture of how sigma factors operate to regulate gene expression in bacteria.

**Figure 4:**
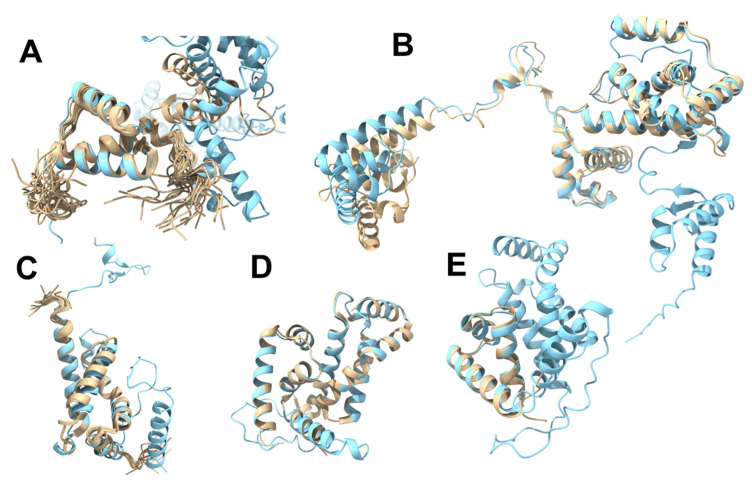
Structural alignments of experimentally solved (partial) *B. subtilis* sigma factors (cream) with the equivalent AlphaFold models (light blue): (**A**) Overlay of the NMR structure of σ1.1 domain of SigA (5MWW) [12] with the AlphaFold model of SigA. (**B**) SigA structure excised from the cryo-EM (7CKQ [23]) structure of the BmrR transcription activation complex [23] overlaid with the full-length AlphaFold model of SigA. (**C**) Overlay of the NMR structure of SigE σ2 domain (5OR5; unpublished) with the equivalent AlphaFold model of SigE. (**D**) 2.6 Å crystal structure of SigW (5WUR [11]) excised from the co-crystal complex with the anti-sigma factor RsiW [11] overlaid with the AlphaFold model of SigW; structured regions are a near perfect match. (**E**) Overlay of the 3.1 Å crystal structure (6JHE [39]) domain bound to the −35 element DNA [39] (hidden) with the AlphaFold model of full-length SigW.

Of those *B. subtilis* sigma factor structures that have been experimentally solved (summarised in Table 1), two were determined via NMR (5MWW [12] and 5OR5 (unpublished)), three were achieved using X-ray crystallography (5WUR [11], 5WUQ [11], and 6JHE [39]), and there was a single available structure of a complex solved by cryo-EM (7CKQ [23]). These systems showcase the relative strengths and weaknesses of each biophysical technique and expose different insights into the respective sigma factors. The NMR structure of the σ1.1 domain from SigA (5MWW [12]) revealed that the domain was unexpectedly compact and, surprisingly, showed similarity to the δ domain of the RNAP [12]. The overlay of this NMR structure also matched well with the AlphaFold model of SigA; the terminal regions of the NMR construct were unsurprisingly heavily disordered, but the core helices of the domain aligned well (Figure 4A). The match between the SigA AlphaFold model and the cryo-EM structure was also good; however, there appeared to be some movement in the cryo-EM structure, which was likely the result of SigA being incorporated into the BmrR-RNAP-DNA complex [23] (Figure 4B). This structure illuminates the sigma factor in its broader context in a way that would likely not be feasible with any other technique. The unpublished NMR structure of the σ2 domain of SigE (5OR5 [12]) was likewise a good match, with the corresponding region in the full length AlphaFold model; however, bigger differences are seen around the turns (Figure 4C). This is fairly unsurprising as these regions are typically modelled with lower confidence by AlphaFold [65], whilst NMR ensembles are well-suited to explore the conformational space of highly dynamic regions. Similarly, the AlphaFold model of SigW overlaps extremely well with the crystal structure of the SigW bound to its anti-sigma factor partner protein, RsiW (5WUR) [11] (Figure 4D). This can likely be explained by AlphaFold being trained predominantly on a library of crystal structures, and so it may be biased towards rigid, well-ordered structures. The crystal structure of the σ4 domain of SigW bound to the −35 region of DNA (6JHE [39]) also mapped well onto the AlphaFold model (Figure 4E).

These few examples of experimentally determined sigma factor structures from *B. subtilis* serve to highlight how well AlphaFold generally handles these highly dynamic systems. This also suggests that the AlphaFold models of those sigma factors lacking experimentally determined structures have excellent utility so long as they are interpreted with caution due to AlphaFold’s propensity to occasionally overbuild helices, most notably observed in the case of SigI (see Table 1). The study of sigma factors will likely also reap the rewards from the ascendancy of cryo-EM, which is better able to peer into more complex and dynamic systems than crystallography. This is exemplified by the cryo-EM structure 7CKQ [23] of the BmrR-RNAP-DNA complex; as time goes on we expect to see many further structures of sigma factors in this DNA-bound context. These combined advances in experimental and computational structural biology will hopefully rapidly translate into corresponding advances in our understanding of bacterial molecular biology.

## Figures and Tables

**Table 2:** Crystallographic parameters.

Protein	SigE
Beamline	Diamond Light Source I03
Data processing	xia2 dials
Resolution Range	63.39–2.379 (2.464–2.379) Å
Space Group	C 2 2 21
Unit Cell	81.953 165.143 98.930 90.00 90.00 90.00
Total Reflections	356785 (16578)
Unique Reflections	27360 (1349)
Multiplicity	13.0 (12.3)
Completeness	100 (99)%
Mean I/Sigma(I)	13.4 (1.8)
Wilson B-factor	48.27
R-meas	0.131 (2.143)
CC1/2	0.999 (0.851)
Reflections used in refinement	27115
Reflections used for R_free_	1332
Final R_work_	0.198
Final R_free_	0.256

## Data Availability

We have deposited the X-ray crystal structure of SigE^17-133^ in the Protein Data Bank in Europe [22] with PDB ID: 8B3Z.
